# Immunogenicity of DNA Vaccine against H5N1 Containing Extended Kappa B Site: *In Vivo* Study in Mice and Chickens

**DOI:** 10.3389/fimmu.2017.01012

**Published:** 2017-08-24

**Authors:** Patrycja Redkiewicz, Anna Stachyra, Róz∙a Sawicka, Katarzyna Bocian, Anna Góra-Sochacka, Piotr Kosson, Agnieszka Sirko

**Affiliations:** ^1^Institute of Biochemistry and Biophysics, Polish Academy of Sciences, Warsaw, Poland; ^2^Faculty of Biology, University of Warsaw, Warsaw, Poland; ^3^Mossakowski Medical Research Centre Polish Academy of Sciences, Warsaw, Poland

**Keywords:** mice, DNA vaccine, kappa B sites, H5N1, influenza, chicken

## Abstract

Influenza is one of the most important illnesses in the modern world, causing great public health losses each year due to the lack of medication and broadly protective, long-lasting vaccines. The development of highly immunogenic and safe vaccines is currently one of the major problems encountered in efficient influenza prevention. DNA vaccines represent a novel and powerful alternative to the conventional vaccine approaches. To improve the efficacy of the DNA vaccine against influenza H5N1, we inserted three repeated kappa B (κB) motifs, separated by a 5-bp nucleotide spacer, upstream of the cytomegalovirus promoter and downstream of the SV40 late polyadenylation signal. The κB motif is a specific DNA element (10pb-long) recognized by one of the most important transcription factors NFκB. NFκB is present in almost all animal cell types and upon cell stimulation under a variety of pathogenic conditions. NFκB is released from IκB and translocates to the nucleus and binds to κB sites, thereby leading to enhanced transcription and expression of downstream genes. We tested the variants of DNA vaccine with κB sites flanking the antigen expression cassette and without such sites in two animal models: chickens (broilers and layers) and mice (BALB/c). In chickens, the variant with κB sites stimulated stronger humoral response against the target antigen. In mice, the differences in humoral response were less apparent. Instead, it was possible to spot several gene expression differences in the spleens isolated from mice immunized with both variants. The results of our study indicate that modification of the sequence outside of the sequence encoding the antigen might enhance the immune response to the target but understanding the mechanisms responsible for this process requires further analysis.

## Introduction

DNA vaccines were introduced over two decades ago. This very promising technique relies on the production of an antigen by the cells of an immunized host after the introduction of a genetically engineered expression cassette for this antigen and, as a consequence, induction of humoral and cellular immune responses (1). Despite multiple advantages, the DNA vaccines have apparent pitfalls that need to be addressed before being applied for veterinary or clinical purposes (2). One of the factors limiting the effectiveness of DNA vaccine is the transport of the plasmid DNA to the nucleus through the nuclear envelope. The links between the transfer of non-viral DNA to the nucleus and the transgene expression level has been mostly explored in relation to therapeutic purposes such as gene therapy and anticancer vaccines [for reviews, see Ref. (3–6)]. Recently, it was suggested that various proteins containing a nuclear localization signal might play a role in the intracellular trafficking of plasmid DNA to the nucleus and that the microtubule network and motors might be required for this process (7). This suggestion is in agreement with earlier observations that either covalent attachment of nuclear proteins to the plasmid or the presence of *cis*-binding sites for nuclear proteins, such as transcription factors, in the DNA of the transfected plasmid might improve the efficiency of non-viral gene transfer (8, 9).

The most promising approaches involve the use of NF-κB (10–12). The transcription factors of the NF-κB family contain an evolutionarily conserved region at their N-terminus named the Rel homology domain (RHD), which is responsible for dimerization and binding to the kappa B (κB) motif in DNA (13–15). Inactive NF-κB dimers are sequestered in the cytoplasm because of their association with the inhibitor of NF-κB (IκB). Upon stimulation, IκB is phosphorylated by the action of a specific IκB kinase, ubiquitinated, and degraded in the proteasome. The released NF-κB dimers enter the nucleus, bind to κB sites in the promoters of the target genes, and initiate (or enhance) their transcription (16). The observation that the κB motif (5′-GGGGACTTTCC-3′) can augment nuclear entry of modified vectors and increase the expression level of reported genes in various transfected human cells was first published in 2001 (12). Similar experiments were later performed by other researchers, who observed positive correlations between the number of κB sites (i.e., 5, 10, or 20 tandem repeats) upstream the cytomegalovirus (CMV) promoter and the transgene expression level in transfected cells of the murine carcinoma colon 26 line (17). Other researchers designed DNA vectors with several κB sites separated by the optimized 5-bp spacer (5′-AGCTG-3′) and used luciferase as a reporter protein to monitor *in vivo* the influence of these sites on the level of transgene expression (10, 11). They found higher luciferase activity in mice transfected with the plasmid containing the expression cassette for luciferase flanked by the sequence consisting of three repeated κB sites separated by spacers than in mice transfected with the plasmid containing the same cassette but without κB sites. Understanding of the mechanisms responsible for the higher expression of transgenes flanked by κB sites in comparison to transgenes without such motifs could help in the translation of this observation into knowledge needed for the purposes of the effective DNA vaccine development.

Influenza virus is an important pathogen, causing seasonal and sudden pandemics in humans and its avian variants can be devastating for domestic poultry. Moreover, zoonotic transmission of highly pathogenic avian influenza viruses, like H5N1, has been reported regularly. Vaccination is the most promising strategy to control the virus, but traditional vaccines are not very useful in the case of new emerging strains, and therefore, the development of innovative, new-generation vaccines is an urgent need.

We have previously published several papers describing our work on development of DNA vaccine against H5N1 influenza (18–20). In this work, we tested two types of DNA vaccine, one containing the κB sites (in triplicates; with 5-bp spacers) flanking the antigen expression cassette and the other without such sites, in two animal models, chickens and mice. Both plasmids had the same antigen expression cassette encoding full-length hemagglutinin (HA) from influenza virus strain A/swan/Poland/305-135V08/2006(H5N1). Experiments with chickens, the natural host of avian influenza virus, focused on the comparison of humoral responses in sera. In mice, not only the humoral response in serum, but also the level of cytokines secreted by splenocytes and the population of cytotoxic T cells (Tc) and helper T cells (Th) were examined. Moreover, the spleen transcriptome profiles of the selected mice groups were examined. The results indicated that in chicken the humoral response was slightly better after immunization with the plasmid containing the κB sites than with the plasmid without such sites. In mice, the difference was much less apparent.

## Materials and Methods

### Plasmid Construction

The K3/pCI plasmid, containing the full-length cDNA of HA from the highly pathogenic influenza virus strain A/swan/Poland/305-135V08/2006(H5N1, clade 2.2), has been described before as a long variant of DNA vaccine (18). The 3NF/pCI plasmid contains the same expression cassette, which is additionally flanked by 45-bp sequences. One is located upstream of the CMV promoter, and the second is located downstream of the SV40 late polyadenylation signal. Each 45-bp sequence contains three repeats of NF-κB-binding sites (5′-GGGACTTTCC-3′) separated by the optimized spacer (5′-AGCTG-3′). The NFGK/pCI plasmid consists of the identical 45-bp sequences but they flank the cassette with the modified cDNA sequence of HA, present in previously described GK/pCI plasmid (20). The plasmids were propagated in *Escherichia coli* (DH5α strain) and isolated using the NucleoBond^®^ PC 10000 EF giga-scaled purification kit (Macherey-Nagel, 740548). Before immunization, the plasmid DNA, suspended in PBS, was mixed with Lipofectin^®^ (18292-037, Invitrogen™) in a 6:1 ratio (DNA:Lipofectin [v/v]), as described before (18).

### Immunization of Animals and Ethic Statement

#### Chickens

Broiler chickens Ross 308 and layer chickens Rosa 1 were purchased from a local commercial brooder on the day of hatching and were maintained at an experimental poultry house under standard bedding conditions. Animals were fed once a day and had free access to water. At the end of the experiment, the animals were humanely euthanized. Chickens were immunized intramuscularly with 60 µg of plasmid in final volume of 100 µl and blood samples were collected from the wing vein. Two doses of vaccine were administered at 7th and 21st days of life.

#### Mice

Specific pathogen-free BALB/c female mice were maintained at the experimental facility at the Mossakowski Medical Research Centre, Polish Academy of Sciences (Warsaw) under a 13-h light/11-h dark cycle with free access to water and standard mouse diet. All groups were immunized intramuscularly with 20 µg of plasmid in final volume of 50 µl and the blood samples were collected from a left ventricle of heart. Two doses of vaccine were administered at 35th and 49th days of life. The schedule of chickens and mice immunization experiments is summarized in Table 1.

**Table 1:** Details of the immunization experiments.

Animal model	Experiment Nr (chicken type; dose)	Group	Size (*n*=)	Days of treatments^a^		
				Immunization	Blood collection	Spleen collection
Chickens	Experiment 1 (layers; 60 µg)	K3/pCI	6	7, 21	21, 28, 35	–
		3NF/pCI	6			
		pCI	2			

	Experiment 2 (broilers; 60 µg)	K3/pCI	10	7, 21	21, 35	–
		3NF/pCI	10			
		pCI	4			

	Experiment 3 (layers; 60 µg)	K3/pCI	10	7, 21	35	–
		3NF/pCI	10			
		NFGK/pCI	10			
		pCI	3			

Mice	Experiment 1 (20 µg)	K3/pCI	6	35, 49	49, 56, 63	63
		3NF/pCI	7			
		NFGK/pCI	7			
		pCI	2			

	Experiment 2 (20 µg)	K3/pCI	8	35, 49	49, 56, 63	63
		3NF/pCI	8			
		NFGK/pCI	8			
		pCI	7			

	Experiment 3 (20 µg)	K3/pCI	3	35, 49	47, 52	52
		3NF/pCI	3			
		pCI	2			

*^a^The numbers refer to the day of life of the animals*.

All efforts were made to minimize suffering. The experiments with chickens were approved by the Second Local Ethical Committee for Animal Experiments at the Medical University of Warsaw, Permit Number 17/2009. The experiments of mice immunization were approved by the Fourth Local Ethical Committee for Animal Experiments at the National Medicines Institutes, Permit Number 03/2014.

### ELISA and Antibody Titers

#### Chickens

The ELISA was performed as described earlier (18). Shortly, the MediSorp Surface (Nunc, UK) plates were coated with 300 ng of the recombinant homologous H5 HA antigen (obtained in baculovirus system; Oxford Expression Technologies, UK) and the bound IgY were detected with goat anti-chicken IgY (Fc-specific)-HRP (Pierce/Thermo Scientific, USA). For the determination of IgY endpoint titers, twofold serial dilutions (in a range of 10^−3^–10^−6^) of chicken sera collected on day 35 were analyzed using the ELISA protocol. The absorption curves were made using Gen5 Data Analysis Software (BioTek Instruments, Inc.), and the highest dilution giving a specific positive result was determined for each serum. The reciprocal of such dilution was defined as the endpoint serum titer.

#### Mice

Sera from immunized mice were tested for antibodies directed against homologous H5 HA by a one-dilution indirect ELISA using MaxiSorp Surface (Nunc, UK) plates coated with 300 ng of recombinant H5 HA (obtained in the baculoviral system; Oxford Expression Technologies, UK). Alkaline phosphatase-conjugated goat anti-mouse IgG (Sigma-Aldrich) was used as the secondary antibody.

### Hemagglutination Inhibition (HI)

Hemagglutination inhibition tests were performed according to the OIE standard procedures using the heterologous hemagglutinating antigen prepared from the low pathogenic H5N2 strain A/chicken/Belgium/150/1999 (DG Deventer, Netherlands). The 25-µl aliquots of serial twofold dilutions (from 1:8 to 1:512) of sera in PBS were added to an equal volume of HA antigen containing four HA units. After incubation (25 min) in V-bottom microtiter plates at room temperature (RT), 25 µl of a 1% suspension of chicken red blood cells was added and incubated for 25 min at RT. HI titers are defined as the reciprocal of the highest dilution of sera that completely inhibited hemagglutination.

### Cytokine Production by Mouse Splenocytes and Percentage of Tc and Th Cells in Spleens

Immunized and control mice were euthanized 2 weeks after the boost dose (day 63) and their spleens were harvested. For determining the cytokine production and percentage of Tc and Th, splenocytes were prepared from spleen isolated from three randomly selected mice immunized in Experiment 2. The spleen cells suspensions were washed in RPMI-1640 medium (Sigma-Aldrich) and treated for 5 min with lysis buffer (Becton-Dickinson, Franklin Lakes, NJ, USA) in order to remove red blood cells. To determine the levels of cytokines in culture supernatants (Experiment 1 and Experiment 2), the cells (2 × 10^6^ per well) were incubated in 96-well plates (Corning, NY, USA) in complete RPMI-1640 without any supplement (negative control), with recombinant H5 HA protein (Oxford Expression Technologies, UK) (10 µg/ml) or with concanavalin A (5 µg/ml). Cells were incubated for 72 h (37°C, 5% CO_2_) and centrifuged (10 min, 1,000 rpm, 4°C). The level of cytokines was quantified in the collected supernatants using the Cytometric Bead Array Mouse Th1/Th2/Th17 Cytokine Kit (Becton-Dickinson) according to the manufacturer’s instructions and a FacsVERSE™ flow cytometer (Becton-Dickinson).

For determining the levels of CD8^+^ (Tc), CD4^+^ (Th) cells and their corresponding activated subpopulations: CD25^+^ (Tc), CD69^+^ (Tc), CD25^+^ (Th), and CD69^+^ (Th) splenocytes (1 × 10^6^ per probe) were incubated 30 min on ice with the following monoclonal antibodies (Becton-Dickinson): PerCP rat anti-mouse CD4, FITC rat anti-mouse CD8, APC rat anti-mouse CD25, and FITC rat anti-mouse CD69. After incubation, the cells were washed three times, resuspended in the Stain Buffer (Becton-Dickinson), and examined by flow cytometry using FacsVERSE™ flow cytometer (Becton-Dickinson). The levels of Tc and Th were determined in the Laboratory of Flow Cytometry, Faculty of Biology University of Warsaw.

### RNA Isolation and Microarray Analysis

Microarray expression analysis was performed using the Affymetrix Gene Atlas system according to the manufacturer’s instructions. RNA was isolated from three independent individuals per treatment (K3/pCI and 3NF/pCI) or two independent control individuals (pCI), 3 days after the boosted vaccination; 100 ng of total RNA that passed the initial quality control screen was then prepared for Affymetrix whole transcriptome microarray analysis using the Ambion^®^ WT Expression Kit (4411973). Prepared samples were hybridized to the Affymetrix^®^ Mouse Gene 2.1 ST Array Strip (Affymetrix, Santa Clara, CA, USA). The microarrays were scanned with the Affymetrix GeneAtlas Scanner, and the intensity signals for each of the probe sets were written by Affymetrix software into CEL files. The CEL files were imported into Partek Genomic Suite v 6.6 software with the use of Robust Multiarray Averaging. During this step, a background correction was applied based on the global distribution of the PM (perfect match) probe intensities and the affinity for each of the probes (based on their sequences) was calculated. Then, the probe intensities were quantile normalized (21), log_2_ transformed, and the median polish summarization to each of the probe sets was applied. Qualitative analysis was the performed, e.g., principal components analysis, in order to identify outliers and artifacts on the microarray. After quality check, the three-way ANOVA model using Method of Moments (22) was applied to the data, which allowed us to create lists of significantly and differentially expressed genes between biological variants (with the cutoff values: *p* value <0.05, 1.4 >fold change >−1.4). Functional analysis of the obtained lists of the genes was performed in Ingenuity Pathway Analysis (Ingenuity Systems, Redwood City, CA, USA, http://www.ingenuity.com). The microarray data were deposited in Gene Expression Omnibus repository under the accession number GSE101747.

### Real-time PCR

In order to validate the microarray data, the gene expression level of the selected genes was determined using the same samples of RNA as those used for microarray analysis. The cDNA was obtained from 1.2 µg of total RNA using the Maxima H Minus First Strand cDNA Synthesis Kit (Thermo Scientific) with Oligo(dT)18 primers and was then used as the template in RT-qPCR using Thermo Scientific Luminaris Color HuGreen qPCR master mix (Thermo Sceintific). PCR was performed using the PikoReal™ Real-Time PCR System (Thermo Scientific). Assays contained the cDNA template diluted 20-fold. A 10-min hot-start activation at 95°C was followed by 40 cycles of 15 s of denaturation at 95°C, 30 s of annealing at 60°C, and 30 s of extension at 72°C followed by dissociation analysis (60–95°C). Relative gene expression was calculated according to the 2^−ΔΔCt^ method using TAF8 or PGK1 as the reference gene. The list of used primers is in Table S1 in Supplementary Material.

### Statistical Analysis

Non-parametric tests, such as Kruskal–Wallis (for the comparison of multiple groups) or Mann–Whitney *U* (for the comparison of two groups) that are components of Statistica 12 (StatSoft, Poland), were used for statistical analysis of the results of the following assays: ELISA, HI, cytokines production, and percentage of immune cells. The groups were considered significantly different if at last one of the tests was positive (*p* < 0.05). The significance of the observed changes in RT-qPCR was calculated by REST-MSC (23).

## Results

### Chickens Response to the Tested Variants of DNA Vaccine

**Figure 1:**
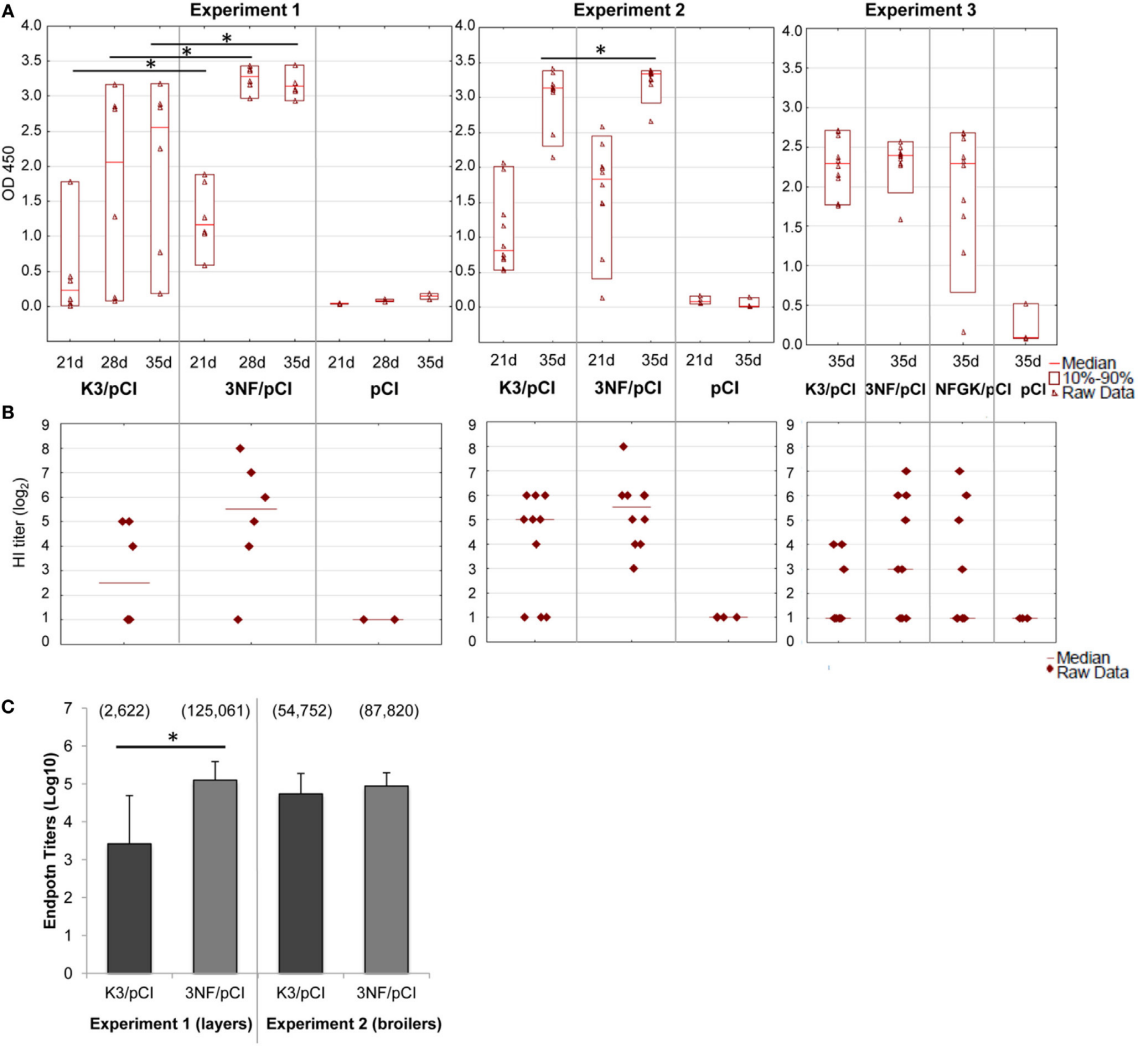
Humoral response of chickens to the tested variants of the DNA vaccine. **(A)** The results of the one-dilution ELISA test shown for individuals with medians and the 10th and 90th percentiles indicated for each group. All sera were diluted 200-fold. Statistically significant differences (*p* < 0.05) are marked by asterisks. **(B)** The individual HI titers and medians in each group (only for day 35) are shown as log_2_. Sera with undetected antibody levels were given an arbitrary value of 1. **(C)** The endpoint titers of anti-H5 hemagglutinin antibodies (day 35) of each group from Experiments 1 and 2. Data are presented as mean values with SD; geometric means are indicated in the brackets. Statistically significant differences (*p* < 0.05) are marked by asterisks.

The effectiveness of the three variants of DNA vaccine against H5N1 was tested first in chickens, the natural host of influenza virus. The 3NF/pCI (and NFGK/pCI used only in Experiment 3) plasmids contained κB sites, while the K3/pCI plasmid did not contain such sites. The variants of the DNA vaccine were tested in chickens in three independent experiments, using either layers or broilers (Table 1). The results of the one-dilution ELISA and the HI tests are shown in Figures 1A,B, respectively. Antibody levels in the 3NF/pCI group detected by ELISA were significantly higher than in the K3/pCI group at all three analyzed time points (days 21, 28, and 35) in Experiment 1 and at one of two analyzed time points (day 35) in Experiment 2. In Experiment 3, the group immunized with NFGK/pCI vaccine was also included. In contrast to the results obtained in Experiments 1 and 2, the differences between groups in Experiment 3 were statistically insignificant on day 35; sera from the other time points are not available (Figure 1A). It is worth to notice that the medians in both groups were lower in Experiment 3 than in the previous experiments, suggesting the overall lower effectiveness of this vaccination trial.

In all experiments, the HI test was performed only with sera collected 2 weeks after the booster (day 35) using an H5N2 commercial antigen. The results shown in Figure 1B indicate that 3NF/pCI groups had always highest median HI than the other groups, although the applied non-parametric tests failed to confirm a statistical significance of the observed difference.

The endpoint titers of anti-H5 HA antibody were determined using sera from the final blood collection (35th day) in Experiments 1 and 2 (Figure 1C). All sera from chickens immunized with 3NF/pCI reached titers above 10^4^ and the majority had titers above 10^5^. Moreover, the highest titer after immunization with 3NF/pCI was 3.4 × 10^5^ and 2.2 × 10^5^ in Experiment 1 (layers) and 2 (broilers), respectively (Figure S1 in Supplementary Material). By contrast, titers in groups K3/pCI were visibly lower and this difference was particularly appreciable in Experiment 1. In Experiment 2, titers of high responders were similar in both groups. The highest titer of K3/pCI was 7.2 × 10^4^ in Experiment 1 and 2.1 × 10^5^ in Experiment 2. In summary, addition of κB sites apparently improved immunogenicity of the vaccine in chicken model.

### Mice Responses to the Tested DNA Vaccine Variants

**Figure 2:**
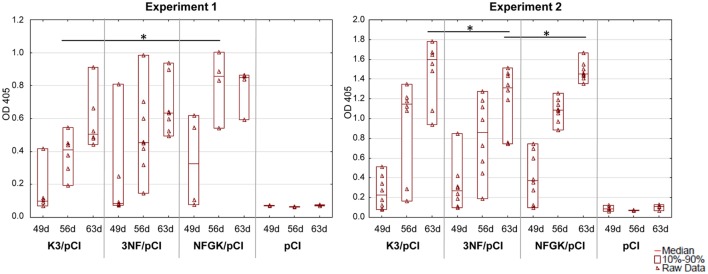
Immune response of mice to the tested variants of the DNA vaccine. The results of the one-dilution ELISA test shown for individuals with medians and the 10th and 90th percentiles indicated for each group, where applicable (Experiments 1 and 2). All sera were diluted 100-fold. Statistically significant differences (*p* < 0.05) are marked by asterisks.

The experiments with mice were performed independently to verify the effectiveness of the three tested variants (K3/pCI, NF/pCI, and NFGK/pCI) of the DNA vaccine. The schedule and other details of the experiments are provided in Table 1. The level of anti-H5 HA antibodies in the sera of immunized mice was monitored by one-dilution ELISA (Figure 2). The values for individuals, medians, and the 10th–90th percentiles are shown for each group at each time point. Although the medians for the NFGK/pCI group were in several cases higher than in the corresponding K3/pCI and 3NF/pCI groups, the statistically significant differences were observed only in Experiment 1 at the 56th day (between K3/pCI and NFGK/pCI) and in Experiment 2 at the 63rd day (between K3/pCI and 3NF/pCI; also between 3NF/pCI and NFGK/pCI).

In order to further explore the differences between the mice groups, the levels of cytokines produced by the stimulated *in vitro* splenocytes (Table 2) and the population of Tc (CD8^+^) and Th (CD4^+^) cells in the spleens (Table 3) have been assessed.

**Table 2:** Cytokine levels produced by stimulated splenocytes isolated from the mice immunized with the tested variants of the DNA vaccine.

	IFNγ (pg)	TNF (pg)
K3/pCI	**1,236** ± 333	**61** ± 32
3NF/pCI	**960** ± 274	**65** ± 30
NFGK/pCI	**964** ± 520	**53** ± 22

*The results are means (in bold) from three individuals (*n* = 3) with SDs indicated*.

**Table 3:** The percentage of cytotoxic T cells (Tc) and helper T cells (Th) and their subpopulations in mice spleens.

DNA vaccine	Percentage of the indicated cells in the pull of isolated splenocytes					
	Tc			Th		
	CD8^+^	CD8^+^CD25^+^	CD8^+^CD69^+^	CD4^+^	CD4^+^CD25^+^	CD4^+^CD69^+^
K3/pCI	**26.9** ± 3.17	**5.3** ± 0.65	**8.2** ± 1.18	**13.8** ± 0.4	**3.9** ± 0.60	**4.1** ± 0.43
3NF/pCI	**25.0** ± 1.15	**4.8** ± 0.15	**7.7** ± 0.25	**13.3** ± 1.37	**3.0** ± 0.40	**3.7** ± 0.40
NFGK/pCI	**26.7** ± 0.65	**5.4** ± 2.26	**13.2** ± 9.00	**13.4** ± 1.40	**5.3** ± 3.76	**5.6** ± 4.00
pCI	**24.4** ± 1.60	**3.9** ± 0.10	**7.8** ± 0.23	**12.7** ± 0.45	**2.8** ± 0.25	**3.3** ± 0.26

*The results are means (in bold) from three individuals with SDs indicated*.

As indicated in Table 2, higher average level of IFN-γ in K3/pCI than NFGK/pCI and 3NF/pCI was observed, while the average level of TNF was similar in K3/pCI and 3NF/pCI and slightly lower in NFGK/pCI. Unfortunately, the differences between the groups did not pass the statistical significance tests. Secretion of IL-2, IL-6, IL-4, IL-10, and IL-17a in all tested group was at the limit of detection (not shown). No secretion of TNFα and a very low secretion of IFN-γ were observed in the culture of stimulated splenocytes isolated from the control group vaccinated with the empty pCI vector (not shown).

As indicated in Table 3, all vaccinated groups had higher average percentage of Tc cells as compared to the control group (pCI). The percentage of Tc CD25^+^ cells was higher in all immunized groups, while the percentage of Tc CD69^+^ was higher in all groups but not in 3NF/pCI. Similarly, all vaccinated groups had higher percentage of Th, Th CD25^+^, and Th CD69^+^, in the spleens than the control (pCI) group. The highest average values of CD69^+^ (13.2%), CD25^+^ (5.3%), and CD69^+^ (5.6%) were in the NFGK/pCI group; however, the large SD values do not allow to qualify these values as significantly different.

**Figure 3:**
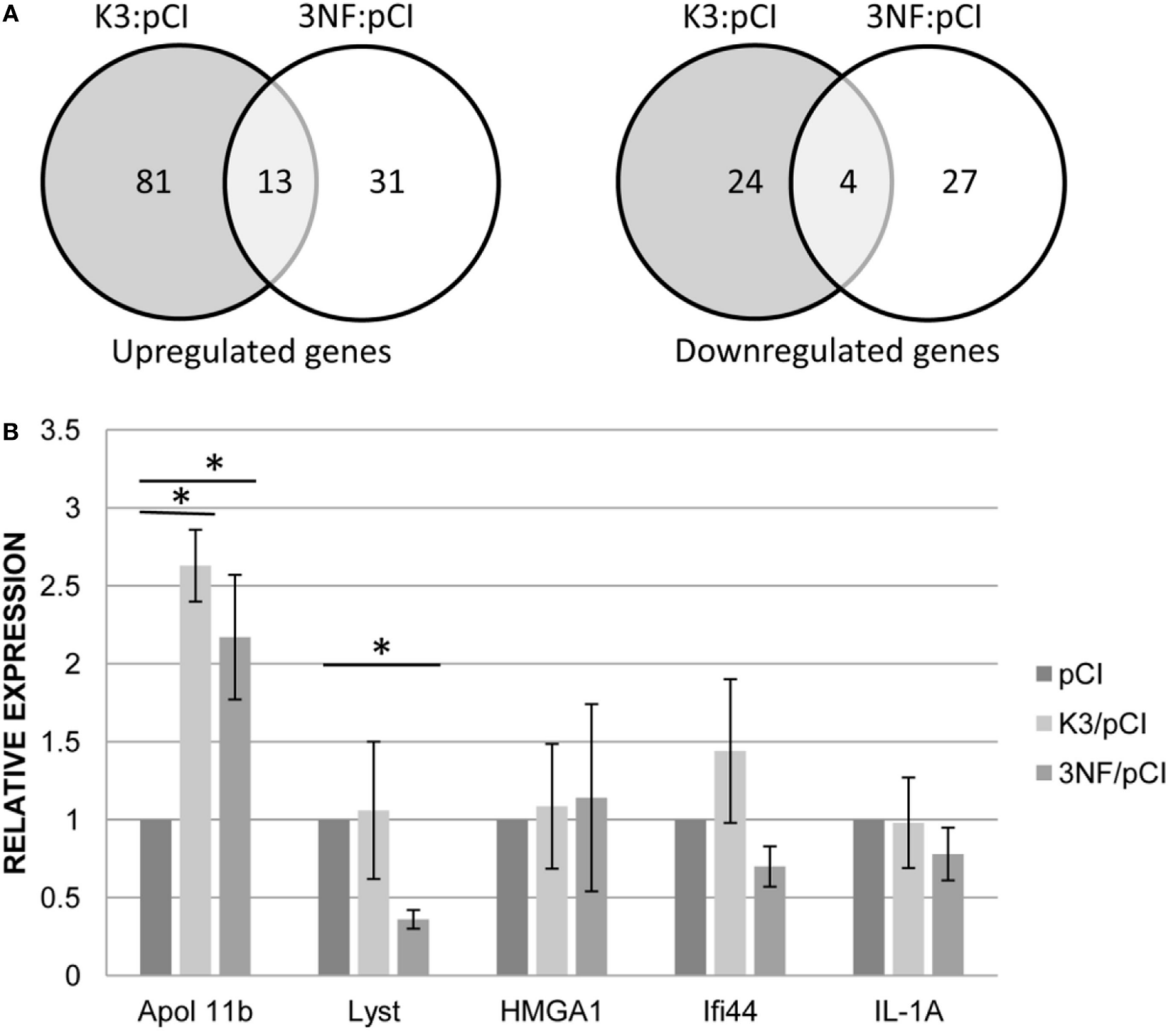
Transcriptomic changes in the spleens of mice immunized with K3/pCI and 3NF/pCI in comparison to the group that received the empty vector (pCI). **(A)** Venn diagrams of upregulated and downregulated genes. **(B)** Results of RT-qPCR for selected genes. The significant changes of the expression (*p* < 0.05) in comparison to pCI group are indicated by the asterisks. Data are presented as mean values of three technical repeats for each sample with SD, calculated for all mice in a group (two mice in the control group and three mice in each experimental group were tested).

More differences between the two variants (K3/pCI and 3NF/pCI) of the tested DNA vaccine were revealed by transcriptional profiling of the mice spleens isolated on day 52 (3 days after the booster) from mice immunized in Experiment 3. The analysis was limited to those 180 genes that had at least a ±1.4-fold difference (*p* < 0.05) in at least one of the groups immunized with the DNA vaccine in comparison to the pCI group (Table S2 in Supplementary Material; Figure 3A). The 3NF/pCI group had an apparently higher percentage (41%) of downregulated genes than the K3/pCI group (23%) and lower percentage of upregulated genes: 59% in 3NF/pCI in comparison to 77% in K3/pCI. Further analysis failed to identify any significantly changed immune-related function or pathway in the selected set of genes. Interestingly, six genes encoding miRNA were upregulated in the K3/pCI group, but only two of them (miR-669o-5p and miR-709) were also upregulated in the 3NF/pCI group. Moreover, miR-181b-1 was downregulated only in 3NF/pCI group, while miR-1186 was downregulated only in K3/pCI. The differences can be also observed in the immunoglobulins transcripts, where few of them were downregulated in K3/pCI (Igkv13-84, Igkv2-112, Igkv3-5, Igkv4-56, Igkv4-74) and none was changed in the 3NF/pCI group (Table S2 in Supplementary Material).

Several genes (Apol11b, Lyst, HMGA1, Ifi44, IL-1A; the last two genes were not significantly changed in microarrays data) were selected for validation of expression by quantitative real-time PCR. The significant differences between the groups were observed only for Apol11b (upregulated in both K3/pCI and 3NF/pCI group) and Lyst (downregulated only in 3NF/pCI). The results of RT-qPCR analysis are shown in Figure 3B.

Summarizing the above results, due to the high individual variability, we do not have strong evidence to conclude that addition of ĸB sites improves the immunogenicity of the vaccine in mouse model.

## Discussion

We demonstrate for the first time in two animal models that the binding sites for NF-κB might improve the efficacy of a DNA vaccine against influenza. It is known that κB motifs can augment nuclear entry of modified vectors and increase the expression level of reported genes in various transfected cells (10–12). The NF-κB proteins and κB sites are evolutionarily conserved and similar signaling pathways are found in mice and chickens (16, 24–26). It has been shown that RHD, p50, is particularly highly conserved between chicken and mammalian, and *in vitro*-synthesized, truncated chicken’s p105 protein (precursor of p50 protein), containing sequences that correspond to the predicted p50 protein, were able to bind the DNA fragment with the consensus κB site in an electrophoretic mobility shift assay (27). Other researchers demonstrated *in vitro* that human NF-κB/Rel-like proteins can bind to the κB-like sequence in the promoter of the gene encoding chicken lysozyme (26). The κB sites used in this work are similar to that found in the promoter of the gene encoding Igκ light chain in human and in the LTR of HIV-1.

Also, numerous studies confirmed that codon optimization to the codon bias of the antigen improves the efficacy of DNA vaccine. Several studies with mammalian cells suggest that increasing the GC content provides better mRNA stability, processing, and nucleocytoplasmic transport. Since we have previously indicated the moderate superiority of GK/pCI vaccine over the K3/pCI vaccine (20), it was of interest to check the effects of the NFGK/pCI variant of vaccine, containing both, the κB motifs and the sequence of HA with the increased percentage of GC in the third position (GC3). One could expect that NFGK/pCI DNA vaccine would be the highly immunogenic. Apparently, we have not observed an anticipated improvement of humoral response after applying of the NFGK/pCI vaccine. Presumably, the large amounts of plasmid DNA or an intracellular protein antigen (translated from the expression cassette provided by the DNA vaccine) might generate too vigorous cellular immune response which kills off the transfected cells too early to maintain immune stimulation, thereby resulting in attenuated vaccine response (28, 29).

The immunological humoral responses induced by the tested plasmids seem to work differently in the used animal models and they slightly vary depending on the experiment. In chickens, the 3NF/pCI worked better than K3/pCI, while a single immunization trial with NFGK/pCI (only in Experiment 3; Figure 1) failed to show its superiority or inferiority (see the above paragraph). The highest ELISA antibody levels were induced by the 3NF/pCI plasmid similarly in three independent immunization trials, although not all experiments gave results that passed rigorous statistical tests verifying differences between the groups that received the plasmid with and without the κB sites. Moreover, the lack of low responders in 3NF/pCI groups suggest that the addition of κB sites could increases the chances of responding to vaccination, what is very desirable. Both, the HI titers and the IgY endpoint titers were higher in the 3NF/pCI groups than in the K3/pCI groups, confirming that the presumed more effective nuclear entry of the modified plasmid can indirectly improve immunogenicity of the vaccine. Furthermore, some differences in the strength and dynamics of the responses observed in different experiments might be attributed to the different genetic backgrounds of the two chicken lines used, as well as their metabolism, growth, and development, which are the results of intensive genetic selection (30). So far, not many studies have been performed on immunological differences between the broiler and layer chicken lines. In the experimental vaccination studies, usually the SPF White Leghorn breed is used, thus this is one of the very first reports studying the humoral response to a DNA vaccine in layer and broiler chickens.

In mice, the differences between the variants of DNA vaccine at humoral level were much less pronounced than in chickens, probably because mice are not the natural host of influenza virus. However, in Experiment 1 (Figure 2), the humoral response in 3NFGK/pCI group is significantly better than that in K3/pCI group on day 56. The subsequent assays, such as monitoring the level of cytokines secreted by stimulated splenocytes (isolated 2 weeks after the booster) and the percentage of Tc and Th population and their CD25^+^ and CD69^+^ subpopulations, might also suggest that the compared variants of the DNA vaccine differentially affect mice immune responses in the spleen. However, high individual variability does not allow us to draw strong conclusions about differences between the immunized groups.

The very low (at the detection limit, not shown) levels of IL-2, IL-6, and IL-10 and absence of IL-4 and IL-17a might be explained by the conditions of the assay and splenocytes cultivation (and induction), which were optimal for IFN-γ, and also by the short half-life of IL-4 (31) and by the high concentration of IFN-γ, which negatively regulates the induction of Th17 cells (32).

Comparison of the transcriptional patterns of splenocytes isolated from mice immunized with the two types of vaccine (K3/pCI and 3NF/pCI) with the transcriptional pattern of the control mice (pCI) revealed some differences between the variants. For example, differences in the transcripts level of two microRNA were detected (Table S2 in Supplementary Material): mir181-b was downregulated in the 3NF/pCI, while mir1186 was downregulated in K3/pCI. Interestingly, mir181-b inhibits the expression of importin-α3 that is crucial for translocation of NF-κB from cytoplasm to nucleus. The level of mir181-b is reduced after proinflamatory stimulation, e.g., by TNF-α and the transcription of NF-κB-dependent genes can be activated (33). Unfortunately, little is known about function of mir1186 but it is suggest that its upregulation might stimulate cell proliferation (34).

Expression of two genes (Apol11b and Lyst) has been positively verified by RT-qPCR. Information about the function of Apol11b and Lyst, two genes with the expression verified by RT-qPCR, is rather limited. The Apol11b gene, upregulated in both tested groups (3NF/pCI and K3/pCI), encodes the Apolipoprotein L variant specific for the spleen (35). Apolipoproteins L share functional similarities with proteins of the Bcl-2 family, taking part in the regulation of apoptosis, and are encoded by genes induced under inflammatory conditions, i.e., type-1 INF. The second gene downregulated in the 3NF/pCI group (but not in the K3/pCI group) was LYST, a lysosomal trafficking regulator. A deficiency in this gene results in the enlargement of lysosomes with abnormal morphology in granulocytes and some other leukocytes (36). Recent data suggest that the LYST protein might be required for the maturation of perforin-containing granules into exocytosis-competent secretory granules (37). Moreover, silencing of the Lyst gene results in the induction of apoptosis in RPMI 8226 myeloma cells (38). Based on these findings, it can be hypothesized that vaccination with 3NF/pCI facilitates and enhances the immune response in comparison to the vaccination with K3/pCI. This is reflected in increased apoptosis, resulting in the removal of immune effector cells that have fulfilled their function and leading to immune response silencing, thereby protecting the vaccinated organism from uncontrolled inflammatory and enabling the preservation of homeostasis (39). This assumption is in agreement with the dynamics of humoral responses, since a substantial increase in induced antibodies was observed earlier in the 3NF/pCI group than in the K3/pCI group.

Despite extensive work on DNA vaccines, reports describing usage of the binding sites for nuclear factors as stimulators of antigen expression and enhancers of the immune response are quite limited. Results of our study highlight possible positive effects of such modifications on the effectiveness of DNA vaccine. However, extended analysis of the mechanisms responsible for the observed effects is needed before such modifications can be put into practice and used for development of highly immunogenic and safe DNA vaccines.

## Ethics Statement

The experiments were approved by the Second Local Ethical Committee for Animal Experiments at the Medical University of Warsaw, Permit Number 17/2009 (chickens) or the Fourth Local Ethical Committee for Animal Experiments at the National Medicines Institutes, Permit Number 03/2014 (mice). All efforts were made to minimize suffering.

## Author Contributions

PR performed mice immunization and responses analysis; ASt performed chickens immunization and responses analysis; RS performed transcriptional analysis; PK involved in mice immunization; KB involved in FACS analysis; AG-S and AS conceived and designed the experiments, and prepared the final version of the manuscript. All authors were involved in writing and reviewing the manuscript.

## Conflict of Interest Statement

Results described in this work are subject of patent application (decision pending). No other conflict of interest declared.

## References

[1] Dufour V DNA vaccines: new applications for veterinary medicine. Vet Sci Tomorrow (2001) 2(2):1–19.

[2] Stachyra A Gora-Sochacka A Sirko A. DNA vaccines against influenza. Acta Biochim Pol (2014) 61(3):515–22. 25210719

[3] Belmadi N Midoux P Loyer P Passirani C Pichon C Le Gall T Synthetic vectors for gene delivery: an overview of their evolution depending on routes of administration. Biotechnol J (2015) 10(9):1370–89. 10.1002/biot.201400841 26037687

[4] Pichon C Billiet L Midoux P. Chemical vectors for gene delivery: uptake and intracellular trafficking. Curr Opin Biotechnol (2010) 21(5):640–5. 10.1016/j.copbio.2010.07.003 20674331

[5] van der Aa MA Mastrobattista E Oosting RS Hennink WE Koning GA Crommelin DJ. The nuclear pore complex: the gateway to successful nonviral gene delivery. Pharm Res (2006) 23(3):447–59. 10.1007/s11095-005-9445-4 16525863

[6] Wagstaff KM Jans DA. Nucleocytoplasmic transport of DNA: enhancing non-viral gene transfer. Biochem J (2007) 406(2):185–202. 10.1042/BJ20070505 17680778

[7] Badding MA Lapek JD Friedman AE Dean DA. Proteomic and functional analyses of protein-DNA complexes during gene transfer. Mol Ther (2013) 21(4):775–85. 10.1038/mt.2012.231 23164933 PMC3616537

[8] Cartier R Reszka R. Utilization of synthetic peptides containing nuclear localization signals for nonviral gene transfer systems. Gene Ther (2002) 9(3):157–67. 10.1038/sj.gt.3301635 11859418

[9] Chan CK Jans DA. Using nuclear targeting signals to enhance non-viral gene transfer. Immunol Cell Biol (2002) 80(2):119–30. 10.1046/j.1440-1711.2002.01061.x 11940112

[10] Breuzard G Tertil M Goncalves C Cheradame H Geguan P Pichon C Nuclear delivery of NFkappaB-assisted DNA/polymer complexes: plasmid DNA quantitation by confocal laser scanning microscopy and evidence of nuclear polyplexes by FRET imaging. Nucleic Acids Res (2008) 36(12):e71. 10.1093/nar/gkn287 18515353 PMC2475639

[11] Goncalves C Ardourel MY Decoville M Breuzard G Midoux P Hartmann B An optimized extended DNA kappa B site that enhances plasmid DNA nuclear import and gene expression. J Gene Med (2009) 11(5):401–11. 10.1002/jgm.1312 19326361

[12] Mesika A Grigoreva I Zohar M Reich Z. A regulated, NFkappaB-assisted import of plasmid DNA into mammalian cell nuclei. Mol Ther (2001) 3(5 Pt 1):653–7. 10.1006/mthe.2001.0312 11356069

[13] Diamant G Dikstein R Transcriptional control by NF-kappaB: elongation in focus. Biochim Biophys Acta (2013) 1829(9):937–45. 10.1016/j.bbagrm.2013.04.007 23624258

[14] Hoesel B Schmid JA The complexity of NF-kappaB signaling in inflammation and cancer. Mol Cancer (2013) 12:86 10.1186/1476-4598-12-86 23915189 PMC3750319

[15] Oeckinghaus A Ghosh S. The NF-kappaB family of transcription factors and its regulation. Cold Spring Harb Perspect Biol (2009) 1(4):a000034. 10.1101/cshperspect.a000034 20066092 PMC2773619

[16] Phelps CB Sengchanthalangsy LL Huxford T Ghosh G. Mechanism of I kappa B alpha binding to NF-kappa B dimers. J Biol Chem (2000) 275(38):29840–6. 10.1074/jbc.M004899200 10882738

[17] Thanaketpaisarn O Nishikawa M Okabe T Yamashita F Hashida M. Insertion of nuclear factor-kappaB binding sequence into plasmid DNA for increased transgene expression in colon carcinoma cells. J Biotechnol (2008) 133(1):36–41. 10.1016/j.jbiotec.2007.08.047 17961782

[18] Stachyra A Góra-Sochacka A Sawicka R Florys K Saczyńska V Olszewska M Highly immunogenic prime-boost DNA vaccination protects chickens against challenge with homologous and heterologous H5N1 virus. Trials Vaccinol (2014) 3:40–6. 10.1016/j.trivac.2014.02.002

[19] Stachyra A Pietrzak M Maciola A Protasiuk A Olszewska M Smietanka K A prime/boost vaccination with HA DNA and *Pichia*-produced HA protein elicits a strong humoral response in chickens against H5N1. Virus Res (2017) 232:41–7. 10.1016/j.virusres.2017.01.025 28159612

[20] Stachyra A Redkiewicz P Kosson P Protasiuk A Gora-Sochacka A Kudla G Codon optimization of antigen coding sequences improves the immune potential of DNA vaccines against avian influenza virus H5N1 in mice and chickens. Virol J (2016) 13(1):143. 10.1186/s12985-016-0599-y 27562235 PMC5000471

[21] Bolstad BM Irizarry RA Astrand M Speed TP. A comparison of normalization methods for high density oligonucleotide array data based on variance and bias. Bioinformatics (2003) 19(2):185–93. 10.1093/bioinformatics/19.2.185 12538238

[22] Eisenhart C The assumptions underlying the analysis of variance. Biometrics (1947) 3(1):1–21. 10.2307/3001534 20240414

[23] Pfaffl MW Horgan GW Dempfle L. Relative expression software tool (REST) for group-wise comparison and statistical analysis of relative expression results in real-time PCR. Nucleic Acids Res (2002) 30(9):e36. 10.1093/nar/30.9.e36 11972351 PMC113859

[24] Chiang HI Berghman LR Zhou H. Inhibition of NF-kB 1 (NF-kBp50) by RNA interference in chicken macrophage HD11 cell line challenged with *Salmonella enteritidis*. Genet Mol Biol (2009) 32(3):507–15. 10.1590/S1415-47572009000300013 21637513 PMC3036038

[25] Munir M Zohari S Berg M Non-structural protein 1 of avian influenza A viruses differentially inhibit NF-kappaB promoter activation. Virol J (2011) 8:383 10.1186/1743-422X-8-383 21810221 PMC3161964

[26] Phi van L. Transcriptional activation of the chicken lysozyme gene by NF-kappa Bp65 (RelA) and c-Rel, but not by NF-kappa Bp50. Biochem J (1996) 313(Pt 1):39–44. 10.1042/bj3130039 8546707 PMC1216906

[27] Capobianco AJ Chang D Mosialos G Gilmore TD. p105, the NF-kappa B p50 precursor protein, is one of the cellular proteins complexed with the v-Rel oncoprotein in transformed chicken spleen cells. J Virol (1992) 66(6):3758–67. 1533881 10.1128/jvi.66.6.3758-3767.1992 PMC241161

[28] Barry MA Johnston SA. Biological features of genetic immunization. Vaccine (1997) 15(8):788–91. 10.1016/S0264-410X(96)00265-4 9234514

[29] Shan S Ellis T Edwards J Fenwick S Robertson I Comparison of five expression vectors for the Ha gene in constructing a DNA vaccine for H6N2 influenza virus in chickens. Adv Microbiol (2016) 6(4):310–9. 10.4236/aim.2016.64030

[30] Buzala M Janicki B Czarnecki R. Consequences of different growth rates in broiler breeder and layer hens on embryogenesis, metabolism and metabolic rate: a review. Poult Sci (2015) 94(4):728–33. 10.3382/ps/pev015 25691756

[31] Conlon PJ Tyler S Grabstein KH Morrissey P. Interleukin-4 (B-cell stimulatory factor-1) augments the in vivo generation of cytotoxic cells in immunosuppressed animals. Biotechnol Ther (1989) 1(1):31–41. 2562642

[32] Harrington LE Hatton RD Mangan PR Turner H Murphy TL Murphy KM Interleukin 17-producing CD4+ effector T cells develop via a lineage distinct from the T helper type 1 and 2 lineages. Nat Immunol (2005) 6(11):1123–32. 10.1038/ni1254 16200070

[33] Sun X He S Wara AK Icli B Shvartz E Tesmenitsky Y Systemic delivery of microRNA-181b inhibits nuclear factor-kappaB activation, vascular inflammation, and atherosclerosis in apolipoprotein E-deficient mice. Circ Res (2014) 114(1):32–40. 10.1161/CIRCRESAHA.113.302089 24084690 PMC4051320

[34] Huang V Place RF Portnoy V Wang J Qi Z Jia Z Upregulation of Cyclin B1 by miRNA and its implications in cancer. Nucleic Acids Res (2012) 40(4):1695–707. 10.1093/nar/gkr934 22053081 PMC3287204

[35] Uzureau S Coquerelle C Vermeiren C Uzureau P Van Acker A Pilotte L Apolipoproteins L control cell death triggered by TLR3/TRIF signaling in dendritic cells. Eur J Immunol (2016) 46(8):1854–66. 10.1002/eji.201546252 27198486

[36] Cullinane AR Schaffer AA Huizing M. The BEACH is hot: a LYST of emerging roles for BEACH-domain containing proteins in human disease. Traffic (2013) 14(7):749–66. 10.1111/tra.12069 23521701 PMC3761935

[37] Sepulveda FE Burgess A Heiligenstein X Goudin N Menager MM Romao M LYST controls the biogenesis of the endosomal compartment required for secretory lysosome function. Traffic (2015) 16(2):191–203. 10.1111/tra.12244 25425525

[38] Bong IP Ng CC Fakiruddin SK Lim MN Zakaria Z. Small interfering RNA-mediated silencing of nicotinamide phosphoribosyltransferase (NAMPT) and lysosomal trafficking regulator (LYST) induce growth inhibition and apoptosis in human multiple myeloma cells: a preliminary study. Bosn J Basic Med Sci (2016) 16(4):268–75. 10.17305/bjbms.2016.1568 27754828 PMC5136762

[39] Czabotar PE Lessene G Strasser A Adams JM. Control of apoptosis by the BCL-2 protein family: implications for physiology and therapy. Nat Rev Mol Cell Biol (2014) 15(1):49–63. 10.1038/nrm3722 24355989

