# Supplementary material for: Immunogenicity of DNA Vaccine against H5N1 Containing Extended Kappa B Site: In Vivo Study in Mice and Chickens
Source: Front Immunol. 2017 Aug 24;8:1012. doi: 10.3389/fimmu.2017.01012 (PMC5573718; doi:10.3389/fimmu.2017.01012)
Supplement: Supplementary file 1 [file table_1.doc]

**Supplementary Table 1.** Primer sequences used for quantitative real-time PCR.

| Gene | Forward primer 5’ – 3’  Reverse primer 5’ – 3’ |
| --- | --- |
| TAF8 (Control 1) | AATCAGCCAGTGACCCCCAAGG  CACGGTATGTCGGAGTTTTGATGTAGG |
| PGK1 (Control 2) | AGCCTCACTGTCCAAACTAGGAGATG  CTGTGGCAGATTCACACCCACC |
| Apol11b | CTCTGGGAGCAACATCATCTGTGAGTAG  GGCTCCAATCAGGTTTCTGGCTTCAG |
| Lyst | CCTGAGCACCACGTGTGTCCAGAT  GCAGTAAAGGGACGATCACAGACTTAGG |
| HMGA1 | GAAAGTCACCACAGCTCCAGGGA  TCACTGCTCCTCCTCAGAGGACTC |
| Ifi44 | atttactgcaggccctgagagactacaa  caataggacccagcagcagaactc |
| Il-1A | agttctgccattgaccatctctctctga  gcaagtctcatgaagtgagccatagc |
