# Supplementary material for: Immunogenicity of DNA Vaccine against H5N1 Containing Extended Kappa B Site: In Vivo Study in Mice and Chickens
Source: Front Immunol. 2017 Aug 24;8:1012. doi: 10.3389/fimmu.2017.01012 (PMC5573718; doi:10.3389/fimmu.2017.01012)
Supplement: Supplementary file 3 [file image_1.pdf]

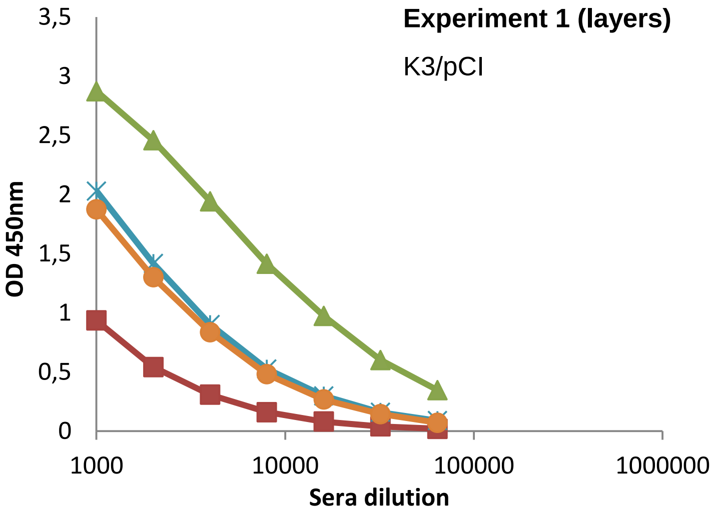

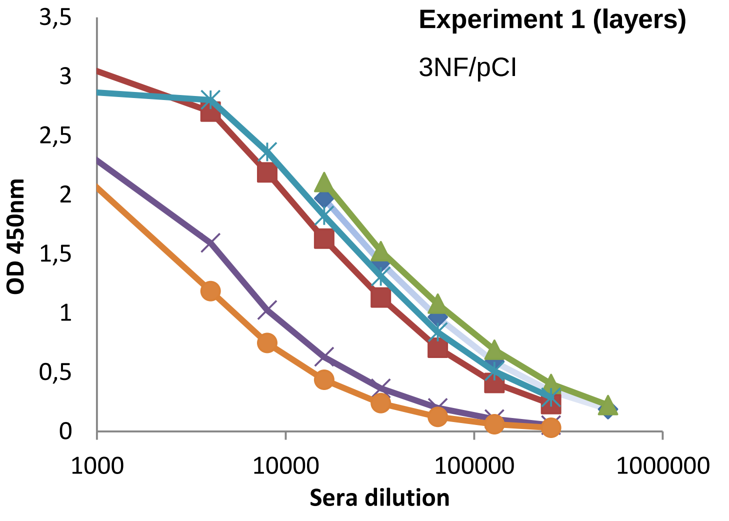

IgY endpoin titers	
K3/pCI	3NF/pCI
37*	283347
3915	178439
72055	349771
155*	38769
14924	233652
13452	23883

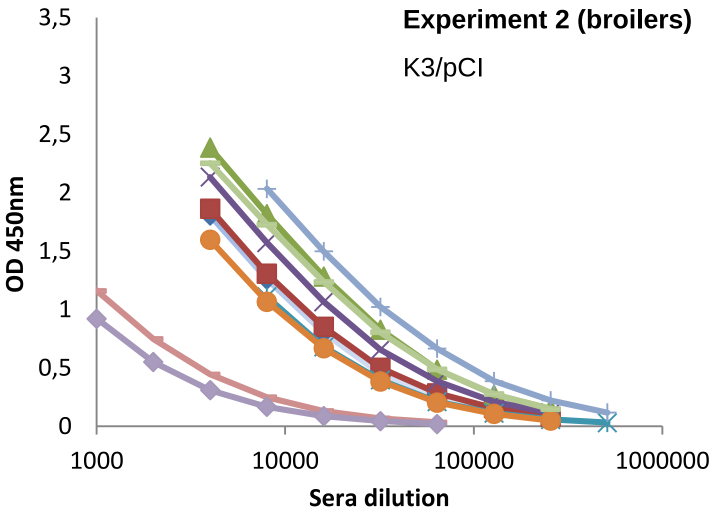

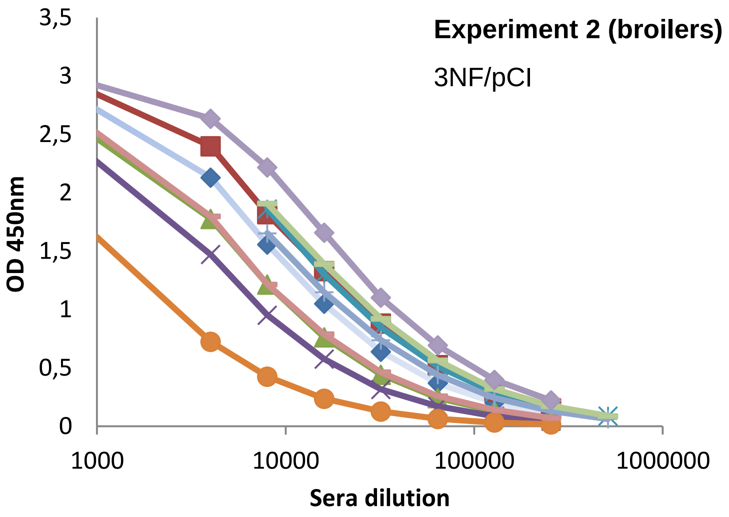

IgY endpoint titers	
K3/pcI	3NF/pCI
63135	100149
73331	149139
139593	61257
105636	41809
53866	149313
51935	15092
219976	123907
7919	66807
142483	169296
5107	225883

Figure S1. Serial dilution ELISA of chicken sera samples. For each individual from Experiment 1 and 2 absorption curves and IgY endpoint titers are shown. Endpoint titer was defined as reciprocal of highest dilution giving a specific positive result , calculated using Gen5 Data Analysis Software (Biotek Inc). * For low responders (OD_{450}<1 at dilution 1:200) serial dilutions wasn't made and the titers were arbitrary determined by multiplying OD value read out by 200.
